# Health and social care professionals' views and experiences of supporting parents with serious mental illness

**DOI:** 10.3389/fpsyt.2023.1284712

**Published:** 2023-12-08

**Authors:** Lucy Oakes, Lauren Wolfenden, Richard J. Drake, Rachel Calam, Lynsey Gregg

**Affiliations:** ^1^School of Health Sciences, University of Manchester, Manchester, United Kingdom; ^2^Greater Manchester Mental Health NHS Foundation Trust, Manchester, United Kingdom

**Keywords:** parental mental illness, adult mental health services, children's services, family, qualitative, template analysis

## Abstract

**Introduction:**

A significant number of individuals with a serious mental illness (SMI) such as schizophrenia or bipolar disorder are also parents of dependent children. Despite the risk of adverse psychological, behavioral, and social outcomes their needs often go unmet. To better understand the needs of parents with SMI and their children it is necessary to gain insight into the perspectives and experiences of the professionals in adult mental health and children's services who work with them, and who, ultimately, are best placed to meet those needs.

**Aims:**

To explore the views and experiences of health and social care professionals working with parents with SMI to understand the needs of, and their role supporting, parents with SMI and their children.

**Methods:**

Semi-structured interviews were conducted with seventeen professionals from six NHS and Local Authority settings in England, UK. Participants were included if they were employed in adult mental health or local authority children's services and had experience of working with parents with SMI. Sampling was purposive, including a wide range of professions in these settings. Interview data were analyzed using template analysis taking a critical realist perspective.

**Results:**

Three top-level themes were generated: (1) Impact of parental SMI on the child, (2) Accessing support from services, (3) Role of professionals working with parents with SMI. Themes highlight diverse, wide-ranging effects of SMI on the child and a reluctance from parents to seek help due to stigma and fear. Available services are reported to be inaccessible and unacceptable to parents with SMI and practitioners experience conflict when balancing the needs of the parent and child. A whole-family approach facilitated by improved communication between services is advocated.

**Conclusion:**

Participants believed that parents with SMI experience complex parenting challenges over and above other parents, describing a largely detrimental impact on the child. Support services were deemed inadequate, and participants stressed the need to develop specialist services tailored toward the needs of parents with SMI and their children. Although participants endorsed joined up working across health and social care settings to facilitate a whole family approach, they required greater service knowledge and training in parental SMI.

## 1 Introduction

Approximately 57–68% of individuals with mental illness are parents of dependent children (1) and by age 16, more than one in four children have experienced living with a parent with mental illness (2). Parents with mental illness are at greater risk of relapse, hospitalization, stigmatization, and social disadvantage than those who are not parents (3, 4) and their children are also at greater risk of a range of poorer outcomes including emotional, social, and behavioral difficulties in childhood. They go on to have poorer mental health in adulthood and experience increased morbidity and premature mortality overall (5–8). The children of parents with the most serious forms of mental illness, such as schizophrenia and bipolar disorder, are at the highest risk of poor outcomes (9). A serious mental illness (SMI) is defined as one which substantially interferes with life activities and often results in severe functional impairment (10).

Caring for children alongside coping with SMI can be difficult for parents. Mental health symptoms and medication side effects may interfere with parents' ability to establish and maintain important family routines (11) and can result in inconsistent and unpredictable parenting (12, 13). Parents with SMI have also been shown to be less emotionally responsive or sensitive to their child's needs (14, 15) although this is not inevitable. Parents with SMI are also frequently rated as displaying “good enough” parenting by clinicians (16). Where positive support networks can contribute to improved recovery and parenting outcomes, negative relationships with wider family can exacerbate parenting stress, stigma of parenting with SMI and feelings of inadequacy (17).

Despite the risk of poor outcomes, children of parents with SMI often remain invisible to services (18). Although there is a mandatory requirement to routinely identify and record parenting responsibilities to dependent children in the United Kingdom (19), a quarter of adult mental health practitioners in adult mental health services fail to do so and fewer still routinely assess children's needs or engage in discussions around parenting (20). Several barriers to the identification and support of families affected by parental SMI have been noted. Adult mental health professionals often lack the knowledge, training, and resources to engage in a more “family-focused” approach (21–23). Many do not see children as being within their remit as adult mental health professionals due to a “patient-centered” approach [e.g., (24)] and believe that other services involved in the family's care should shoulder responsibility for the child (25).

In the UK, separate Childrens' services are typically responsible for providing support to families that are struggling and research shows that 50–90% of parents referred to these services are experiencing mental health problems [Social Care Institute for Excellence; (26)]. Professionals in these services hold responsibility for identifying risks and safeguarding children (27) but do not typically hold mental health qualifications and may feel ill-equipped to support parents with SMI. Poor integration of adult mental health and children's services (28) and a reluctance of parents to seek help due to stigma and fears of custody loss (17, 29) are barriers to effective cross-disciplinary support being provided. Staff in both services may also face difficulties maintaining a dual focus due to conflict between the parent and child's needs (23, 30).

Although parents with SMI often value their parenting role as an important component of their identity and sense of self (16) and incorporating parenting as a focus of recovery-oriented practice within interventions has been found to improve both parental and child wellbeing (31, 32), such interventions are not routinely available in UK services and the needs of parents with SMI and their children often go unmet (33). To gain an in-depth understanding of these needs from a service-level perspective it is necessary to access the views and experiences of the professionals involved in their care (34). These professionals act as gateways to services and interventions made available to parents with SMI and their children (35). Recent qualitative research has started to explore the perspectives of health care professionals in adult mental health services in relation to parents with psychosis (24, 36, 37). However, to comprehensively understand the needs of parents with SMI and their children, and how they are currently supported within services, it is necessary to gain insight into the full range of perspectives from both adult mental health and children's social care professionals, including the perspectives of staff in children's services, who occupy a critical position in identifying and supporting the needs of the child (27).

We utilized individual interviews to explore the views and experiences of a range of health and social care professionals working within NHS adult mental health and local authority children's services. Our aim was to identify the unmet needs of families affected by parental SMI and to understand practitioner perspectives of their role in supporting such families. Template analysis was chosen to facilitate a structured, inductive approach to data analysis (38).

## 2 Methods

### 2.1 Design and ethical approval

A qualitative methodology, using one-to-one semi-structured interviews explored health and social care professionals' views of parents with SMI. Ethical approval was granted by the University of Manchester Research Ethics Committee.

### 2.2 Participants and recruitment

Eligible participants were provided with the participant information sheet. This informed participants that the aim of the study was to explore their experiences of working with parents experiencing SMI and to discuss their role as a care provider. We did not include the term “family-focused” to avoid recruiting professionals who were biased toward family-focused work. Seventeen participants were recruited from three adult mental health services and three local authority children's services across the North and South of England, UK. Purposive and snowball sampling ensured inclusion of a range of professions in each setting, reflecting the multidisciplinary nature of these services. To be included, participants had to be health or social care professionals with at least 6 months of experience providing support to parents with SMI.

### 2.3 Procedure

Interviews were conducted over the telephone to allow access to participants from several settings across a wide geographical area. After providing written consent, participants provided details of their job role, qualifications, employing organization, length of experience working with parents with SMI, as well as their age, sex, and ethnic background.

Interviews were guided by a flexible topic guide which comprised two main sections: (1) views of the parent and child's needs; (2) views of their own role supporting parents with SMI. Prompts and probes were used where appropriate to encourage clarification, elaboration and detail of specific examples or experiences. Interviews ranged from 26 to 70 min in length (mean = 51 min). The interviewer completed a post-interview debrief and reflection to highlight any concerns with the topic guide regarding the phrasing, order, and relevance of questions, and to note whether unique insights were still emerging in relation to the study aims. This informed necessary modifications to the topic guide and confirmed that the data were of adequate quality and richness to allow data sufficiency to be reached (39).

### 2.4 Data analysis

Interviews were audio-recorded with an encrypted device and transcribed verbatim. Identifying information was removed and transcripts pseudonymized prior to analysis. Data were analyzed using template analysis, a form of thematic analysis which provides a systematic approach to data analysis while facilitating the exploration of the richest aspects of data by utilizing a hierarchical coding structure (38). Template analysis involves the generation of a coding template which organizes themes, recurrent elements of participants' narratives, in a meaningful and relational manner (40). The analysis was approached from a critical realist perspective, which combines a realist ontology and relativist epistemology (41).

Data analysis was guided by the procedural steps outlined by Brooks et al. (38). Data familiarization was achieved by transcribing interviews and reading the transcripts. Using NVivo 12, preliminary line-by-line inductive coding was firstly conducted with a subset of five transcripts, which captured a cross-section reflective of the views across the data set and ensured inclusion of a diverse range of professional roles. Semantic coding captured explicitly stated views and experiences, and ensured the analysis remained close to participants' meanings and interpretations (42). Codes were then collated into meaningful clusters which were organized into the first version of the coding template, in which top-level themes and sub-themes were refined and defined. The first version template was then applied to the next subset of five transcripts via an iterative approach. The template was revised and modified where necessary; novel insights were added and two themes were merged due to substantial overlap. The coding template was then applied to the final seven transcripts, and additions and refinements made where necessary. The template was finalized and applied across the entire data set to ensure comprehensiveness. The template was deemed sufficient at the point at which the majority of data relevant to the research question could be coded (38). The final template included three top-level themes each with sub-themes.

### 2.5 Rigor and reflexivity

Interviews were conducted by the second author, and the coding frame was developed, and analysis conducted, by the first. To ensure quality in the analysis (43), the development of the coding template was discussed in regular supervision meetings with a senior team member (LG). This ensured that the analysis was grounded in the data and credible interpretations were formed. LO kept a reflexive log to consider the influence of their own experiences and assumptions on data analysis (44). An audit trail was also established, an important quality assurance technique in template analysis (43). This forms a transparent step-by-step record of the key analytic decisions made during the development and modification of each version of the coding template.

## 3 Results

### 3.1 Sample characteristics

Participants ranged from 24 to 54 years old (*M* = 40.41, *SD* = 8.14). The majority were female (15, 88%) and White British (13, 76.4%). Eleven participants were recruited from adult mental health services (NHS) and six from children's services (Local Authority), reflecting fewer professional roles in the latter. See Table 1 for individual participant demographic information.

**Table 1:** Participant characteristics.

**Participant ID**	**Occupation**	**Setting**	**Experience working with parents experiencing SMI (years)**	Relevant education/ qualification level^*^
1	Nurse specialist	NHS trust	20	Level 5
2	Team leader (family intervention)	Local authority	10	Level 5
3	Social worker	Local authority	10	Level 7
4	Team manager (children's services)	Local authority	15	Level 4
5	Early help practitioner	Local authority	5	Level 3
6	Clinical psychologist	NHS trust	20	Level 8
7	Social worker	NHS trust	13	Level 6
8	Assistant psychologist	NHS trust	6	Level 6
9	Clinical psychologist	NHS trust	12	Level 8
10	Early help practitioner	Local authority	4	Level 3
11	Early help practitioner	Local authority	5	Level 6
12	Occupational therapist	NHS trust	7	Level 6
13	Community psychiatric nurse	NHS trust	4	Level 5
14	Community psychiatric nurse	NHS trust	20	Level 6
15	Social worker	NHS trust	4	Level 6
16	Clinical psychologist	NHS trust	21	Level 8
17	Consultant psychiatrist	NHS trust	17	Level 8

* Level 3 (National Vocational Qualification), Level 4 (Diploma), Level 5 (Diploma), Level 6 (Undergraduate degree), Level 7 (Masters degree), Level 8 (Doctorate).

### 3.2 Themes

Three top-level themes were generated: (1) Impact of parental SMI on the child; (2) Accessing support from services; (3) Role of professionals working with parents with SMI. The final template outlining top-level themes and sub-themes is presented in Table 2.

**Table 2:** Final template of top-level themes and sub-themes derived from the analysis.

**1. Impact of parental SMI on the child**
1.1 A detrimental impact 1.1.1 Consistency, boundaries and routine 1.1.2 Behavioral and developmental implications 1.1.3 Psychological impact and transmission of mental health 1.1.4 Reduced emotional availability and responsiveness 1.1.5 Instrumental and emotional parentification 1.1.5.1 A desire to protect the parent 1.2 Adverse impact is not inevitable 1.2.1 A parent's strengths
**2 Accessing support from services**
2.1 Seeking support 2.2 Barriers to accessing services 2.2.1 Awareness and understanding of services 2.2.2 Practical barriers 2.2.3 Stigma toward parental SMI 2.2.4 Fear of custody loss and social service involvement 2.3 Current services are inadequate 2.3.1 Availability and accessibility 2.2.1 Service user acceptability
**3 Role of professionals working with parental SMI**
3.1 Building the therapeutic relationship 3.2 Child vs. parent as the priority 3.3 Whole family approach 3.4 Communicating information to the child 3.5 Communication and partnership between services 3.6 Staff role limited and influenced by training 3.6.1 Staff require greater understanding of other services and professionals 3.6.2 Lack of training regarding parental mental

#### 3.2.1 Theme one: impact of parental SMI on the child

Practitioners were keen to emphasize that parenting challenges are universal to all parents, expressing the normality of parenting stresses. They noted that outcomes are variable, and detrimental impacts for children are not inevitable, particularly when there is a supportive network around the family allowing the parent to “focus on their own health” (P3, Social Worker, Local Authority).

“Many parents with mental health problems are amazing parents and just because somebody has difficulties with their mental health does not mean they can't care for their kids” (P1, Nurse Specialist, NHS).

At the same time, parents with SMI were described as having parenting challenges over and above those of other parents due to difficulties balancing the dual demands of parenting and coping with the effects of illness and medication. The children of parents with SMI were described as being at increased risk of a range of adverse consequences including “parentification” (the child providing emotional and practical support for the parent), emotional and physical neglect, and the intergenerational transmission of mental illness.

Participants highlighted the impact of early life influences on child development. Symptoms such as agoraphobia, paranoia and withdrawal in the parent were reported to result in fewer opportunities for children to engage in activities outside of the family or home. This was reported to have an impact on the child's early emotional, social, and cognitive development. Parents were often described as emotionally unresponsive, leaving children feeling “unloved, angry, upset and confused” (P9, Clinical Psychologist, NHS): “I guess the issues are about their ability to be emotionally available and also to have the ability to you know of attunement and reciprocity […], the ability to be able to modulate how they respond” (P6, Clinical Psychologist, NHS). Participants reported instances of children being neglected, with parents unable to meet basic needs when illness was acute: “there were no beds or hardly any food or electric” (P11, Early Help Practitioner, Local Authority); “The house is uncleaned, the children's needs are not being met” (P5, Early Help Practitioner, Local Authority).

Participants reported that parents with SMI sometimes experience difficulties setting boundaries for children and in providing consistent parenting: “when you're really really depressed or really anxious or really psychotic or whatever, you are very very difficult to set boundaries in place” (P1, Nurse Specialist, NHS). Parenting practices were described as unpredictable, and dependent upon the parent's fluctuating mental health. This unpredictability was believed to generate uncertainty for the child whose feelings of safety and security may be compromised as a result: “[the parent] starts to behave bizarrely and it could become very frightening […], that has been terrifying for the children” (P9, Clinical Psychologist, NHS).

“It may be that the parent has bipolar affective disorder and it's fine most of the time but then will suddenly become depressed, so the child goes from having a parent that's there and supportive and fine to suddenly parent's crying all the time, and they've no idea what's going on or what's happening” (P17, Consultant Psychiatrist, NHS).

Practitioners reported that inconsistent and permissive parenting can arise due to feelings of guilt: “they almost overcompensate and can become quite indulgent to their children so you end up with all sorts of chaotic behaviour that spirals because the children then feel unsafe and don't know boundaries” (P6, Clinical Psychologist, NHS).

When parents lacked insight into their illness, they were described as not sufficiently able to separate themselves from their psychotic symptoms and delusional beliefs to adequately consider the child's perspective or feelings. Observing a parent experience such symptoms was believed to have adverse behavioral and psychological implications for the child, with parent and child mental health reported to go “hand in hand” (P2, Family Intervention Team Leader, Local Authority):

“The father in that household has got quite severe mental health issues and quite frequent psychotic episodes etcetera and the children's behaviour, one of the insights he's come out with is that the children's behaviour will often mirror his behaviour so they withdraw or they explode” (P2, Family Intervention Team Leader, Local Authority).

In addition to these shorter-term effects, practitioners also highlighted long-term implications for children including the intergenerational transmission of mental illness: “you've got an elevated risk for having mental health problems yourself since you've got a parent who have” (P16, Clinical Psychologist, NHS).

Participants noted that children often faced pressures and responsibilities beyond their peers. They mentioned the occurrence and impact of instrumental and emotional parentification whereby the child adopted the role of a young carer for their parent. Children were reported to be responsible for tasks typically identified as not age-appropriate including paying for utilities, caring for siblings, and managing their parent's medication. Children were also reported as sometimes being a source of emotional support to their parent which descended from feelings of responsibility for their parent's wellbeing and safety: “[children] feel the need to be fixing the parent that is not so well, from in some way it's their fault that they take on this kind of real emotional responsibility for the adult” (P1, Nurse Specialist, NHS). Participants believed that children often felt protective of their parents and made conscious efforts to conceal any difficulties their parent was experiencing in fear of social service involvement and being “taken away.”

Some participants believed that parents often over-relied on the child to maintain their wellbeing, particularly where the parent's needs were not met by services. Feeling burdened with the responsibility of a parent's care was described to have poor social outcomes for the child: “their social life isn't that brilliant because they're worried about mum, their worries escalate and obviously has a big impact on their health and social wellbeing” (P5, Early Help Practitioner, Local Authority). Children were reported to hide their parent's illness from others due to indirect consequences of parental SMI such as social stigma, bullying, and feelings of shame and embarrassment: “the stigma side of things you know at school peer groups […], young people being teased or bullied if they've got a parent with a mental health problem they could be ashamed” (P16, Clinical Psychologist, NHS).

Although many parents were described as preoccupied with their mental illness at the expense of parenting responsibilities, some practitioners highlighted the importance of recognizing a parent's strengths. Lived experience of mental illness was reported to increase compassion in some parents. They were reported to be mindful of their child's wellbeing and displayed empathy toward the child's experiences. Practitioners noted that some parents actively sought to protect their child from adverse experiences and ensured their child remained a priority: “regardless of what they're going through with their mental health, their child will be their sole focus […] they will do everything in their means to make sure their child is protected” (P12, Occupational Therapist, NHS). For some parents, their identity as a parent was described as a being a protective factor in their wellbeing. Children were viewed as a source of motivation and focus for recovery: “If it wasn't for that child, this person may not be with us or may be a lot worse than they already are […] I've got to be strong for my child” (P12, Occupational Therapist, NHS).

#### 3.2.2 Theme two: accessing support from services

This theme captured participants' accounts of parents' help-seeking behavior in relation to both adult mental health and children's services and outlines perceived barriers to accessing support. Practitioner views of the services available for parents with SMI are also included.

Participants reported that most parents with SMI were reluctant to seek support from services and would often “wait until things become virtually crisis point before they seek help” (P14, Community Psychiatric Nurse, NHS). Practitioners attributed this reticence to a lack of insight into their mental health and not perceiving the need for support: “some people don't think they've got mental health issues, some don't want to recognise it” (P4, Children's Services Manager, Local Authority). For many parents, seeking mental health support was not recognized to be a priority, particularly when balancing parenting responsibilities.

Help-seeking was additionally hampered by a lack of awareness and understanding of the services available: “parents aren't sure where to go for help” (P8, Assistant Psychologist, NHS); “you can't go if you didn't know it existed” (P1, Nurse Specialist, NHS). For parents already involved with services, many were described as struggling with ongoing engagement as they did not consistently attend appointments. Practical barriers to engagement included the location of services and the financial burden of transport and childcare. Symptoms of mental illness such as paranoia and agoraphobia were also reported to affect parents' ability to attend appointments.

A key factor in reluctance to seek help was perceived stigma. Practitioners reported that parents feared blame and judgement of their parenting abilities from professionals and wider society: “there's the worry that if they're having difficulties that other people will ultimately judge them and say that they're not good enough parents because they've got mental health problems” (P17, Consultant Psychiatrist, NHS). Stigma was described to be particularly salient among families from cultural backgrounds in which mental illness is perceived to bring shame to the family: “it's normally kind of frowned upon, as failing, getting help from outside agencies” (P17, Consultant Psychiatrist, NHS). Participants also noted a particular reluctance to seek help among fathers with SMI who believe “it's a weakness to ask for help” (P2, Family Intervention Team Leader, Local Authority). This coincides with recognition of a disproportionate emphasis on maternal mental health and a lack of support targeted toward fathers. Professionals highlighted the value of normalizing parenting difficulties amongst these groups to reduce the stigma attached to seeking support. Connecting with other parents and sharing parenting experiences was seen as a valuable tool to reassure parents that parenting challenges were common for all parents, including those without mental illness.

Stigma toward parenting with a SMI was reported to exacerbate fear of social service involvement and subsequent custody loss of children, which was reported by most participants to be a major barrier to parents with SMI seeking support: “that's everyone's number one fear - that their kids will be taken away” (P15, Social Worker, NHS). For parents already involved with social services, there was a perception of increased pressure to meet high expectations and present as the “perfect parent”: “I meet a lot of parents who are striving so hard to be perfect ideal parent because they want to overcompensate because they feel like they're under scrutiny and stuff and actually that puts, it's so much pressure on them and they always feel that they're failing” (P9, Clinical Psychologist, NHS). However, professionals identified that their ultimate goal was to prevent custody loss by putting support in place for parents and keeping families together.

When asked to discuss the services available to parents with SMI, most participants referred to their inadequacy. Services were reported to be struggling due to funding cuts, which had resulted in fewer appointments and increased waiting lists. Without early intervention, mental health and parenting difficulties were described to escalate, having a detrimental impact on both parents and children: “I think it's kind of getting there early really ‘cause most of the time the services are not there and it's gone to the point where it's too late for the parents, and for the children” (P11, Early Help Practitioner, Local Authority).

Participants believed that parents did not always feel listened to or accepted by healthcare professionals. They reported that some parents had poor experiences with General Practitioners (GPs) who were deemed unequipped to deal with complex mental health problems, lacked knowledge of referral pathways and were often dismissive of mental illness. Due to time-limited appointments, participants believed parents refrained from openly sharing any difficulties: “with the GP they only have that 10-minute slot but most of our parents with mental health are guarded and it takes a while for them to discuss what it troubling them” (P11, Early Help Practitioner, Local Authority).

Participants highlighted the lack of, and need for, specialist services dedicated to parents with SMI. They noted that parents often did not meet the criteria for existing services and fell into a gap without support: “[parents] are falling between these two extremes if you like very low level attend your GP very high level come in for some treatment, and there's a bit in between that there's nothing for” (P2, Family Intervention Team Leader, Local Authority). When support was provided, the time-limited sessions of psychological intervention were deemed insufficient and there was a lack of continuous, long-term provision: “one hour a week is not going to fix somebody, there's a hundred and sixty something hours in a week you know what you do on those other days too” (P1, Specialist Nurse, NHS). They perceived that this was partly due to a lack of trained professionals to provide appropriate parenting or mental health support.

Overall, many participants reported the need for flexibility within services depending on the parent's level of need and highlighted the importance of a “bespoke tailored approach” (P2, Family Intervention Team Leader, Local Authority).

#### 3.2.3 Theme three: role of professionals working with parental SMI

This theme captured participants' views toward their role as a health or social care professional working with parents with SMI and their families. It was clear that participants experienced conflict between prioritizing the parent and child's needs and advocated for a whole family approach. Participants also discussed how their role was limited by their knowledge of other services and lack of training to support parents with SMI.

Many participants emphasized the initial importance of building a therapeutic relationship with the parent to improve engagement and described creating a safe space to allow parents to share openly and honestly: “it's the way in which we work with someone, so they feel comfortable talking to you […] having that safe space” (P9, Clinical Psychologist, NHS). Professionals discussed implementing skills such as non-judgemental listening, being empathic toward parents, and recognizing and valuing parents as experts by experience: “no actually you're still not the expert [parents] are, they live it 24/7” (P2, Family Intervention Team Leader, Local Authority).

However, participants expressed difficulties balancing the therapeutic relationship with the parent while managing their professional responsibilities and safeguarding duties toward the child. Some believed it was particularly difficult for social workers to establish trusting and engaging relationships with parents due to their prevailing fear of custody loss. Participants therefore believed it was important to be transparent to parents about their role: “it's about how you have those conversations, how you're very clear from the start around confidentiality and your professional responsibility” (P13, Community Psychiatric Nurse, NHS).

Although building a therapeutic relationship with the parent was important to encourage engagement with services, some participants, including some of those working within adult mental health services, believed the children of service users were their ultimate priority: “absolutely the adult and the parent with the mental illness is massively important but the little people in that house are more important” (P1, Specialist Nurse, NHS). Some participants therefore described adapting the focus of their work to incorporate the child's needs. Communicating with children to support their understanding of their parent's illness and the services involved in their care was considered important.

Without sufficient communication and transparency about their parent's illness, children appeared to face detrimental consequences: “if the kids don't get a narrative for what's going on I think that can be very damaging to them [...] without giving kids language and a framework to understand that I think the consequence of that can be long-lasting” (P16, Clinical Psychologist, NHS). This participant feared that if information was absent, the child would compensate by “filling those gaps in with their own imagination and come up with a worse story” (P16, Clinical Psychologist, NHS).

However, there were concerns that within social services, the parent's needs may be overlooked as a result of focusing on the child: “social services kind of miss the parent's needs and I can see why they do that ‘cause they've got to look at risk and the children's needs and they've got to put the children's needs first but then you've got to kind of balance a little bit of everything” (P8, Assistant Psychologist, NHS). Many participants emphasized the importance of considering both perspectives and therefore discussed the value of working holistically toward a whole family approach: “not only you have to take [parent's] needs into account but we have to be thinking about the needs of the rest of their family and most specifically their children” (P1, Specialist Nurse, NHS).

Professionals expressed the value of joined up working within health and social care services to co-ordinate care for the parent, child, and wider family. Although participants felt skilled within their own service, they believed that a parent's care would improve following collaboration with other services. They discussed forming a partnership with other agencies to develop a shared formulation which captured the needs of the whole family.

“I love the idea of sort of joint working going on between health visiting teams and within schools and you know within modalities as well like peer and secondary care. I've done some joint working with a few families […] I think it sped up kind of positive change within the family” (P16, Clinical Psychologist, NHS).

Although professionals advocated for a joined-up approach, some felt they required greater service knowledge and understanding of the role of other professionals to improve the whole family's care. They stressed the need for training regarding the referral pathways and eligibility criteria for services to recommend appropriate support. One participant from adult mental health services felt they lacked knowledge about support services available to parents with SMI and their children: “I would have no idea what, the only thing I could do is if they were struggling was refer them to social services, but that's not ideal, but you know, I wouldn't know what's available” (P17, Consultant Psychiatrist, NHS). Conversely, professionals in children's services felt unequipped to support parent's mental health needs. Both health and social care professionals discussed the limitations of receiving only generic mental health training and felt they required specialized training to support specifically parents with SMI.

“I've had to work out as I go along from my experience of working with families. And then when they've said to me that they've got a certain mental health condition, I've then gone back and looked it up on the internet […] I've gained more experience and understanding of it, from my own interests” (P10, Early Help Practitioner, Local Authority).

## 4 Discussion

The aim of this qualitative study was to explore health and social care professionals' views of, and experiences supporting, parents with SMI. Participants demonstrated that parents with SMI are balancing the dual demands of mental health and parenting, which appeared to be influenced by factors such as their support network and medication side effects. All participants reported that parental SMI could have a detrimental impact on the child, however some highlighted variability in outcomes and stated that an adverse impact was not inevitable. Participants also discussed barriers to accessing parenting and mental health support such as stigma and the fear of custody loss. To engage parents in services, participants highlighted the value of building a therapeutic relationship with the parent. Participants identified the importance of recognizing the child's needs and advocated for a whole family approach, although recognized the need for further training in parental SMI and improved collaboration between agencies.

This study supports research indicating that although many stresses are universal to all parents, parents with SMI can experience complex parenting challenges over and above those of other parents (4, 45). Consistent with previous research, participants believed that mental health and parenting were intertwined and referred to parents as balancing dual demands (46). In line with existing research, participants indicated that the relationship between parenting stress and mental health was bi-directional and cyclical (30, 47–49), highlighting the need for professionals to identify and support parenting stressors. Although the parenting role was typically perceived to be a source of stress, participants highlighted that for some, their identity as a parent was a source of motivation for recovery. Parents have reported valuing their parenting role which provides a sense of self, meaning and purpose, and hope about the future (12, 50). This reinforces the value of incorporating parenting into interventions as a central component to recovery (32).

The impact of parental SMI on the child is widely discussed in the literature and became a prominent theme in this analysis. Consistent with previous research, participants believed that symptoms of SMI resulted in unpredictable and inconsistent parenting (13) which created an environment lacking in safety and security for the child (51). They reported that witnessing psychotic symptoms was distressing for children, particularly if the child lacked understanding of their parent's illness. In line with participants' accounts, previous research argues that providing children with education about parental mental illness reduces self-blame and guilt, ameliorates their misconceptions, and provides children with appropriate language to communicate their experiences (52, 53). It is therefore essential that professionals communicate information to support the child's understanding of parental SMI (54) and support families in their discussions of parental mental illness (53). Participants also highlighted the incidence of parentification (36). Young carers face an increased risk of early mortality and psychiatric morbidity (55). However, as recognized by participants, young carers are not always visible. Parents and children were reported to conceal difficulties to protect themselves from social service involvement. Professionals must therefore be particularly mindful and perceptive of any signs of adversity for the child to provide early support.

As previous literature highlights (36), most participants focused on the negative elements of parenting with SMI. However, some emphasized variability and in support of previous research, believed that SMI did not reliably predetermine inadequate parenting or detrimental outcomes for the child (16). Participants reported that some parents were sensitive to the child's wellbeing, exhibiting compassion and empathy derived from their lived experience of mental illness. Children of parents with mental illness further describe themselves as more independent, resilient, and empathic than their peers (56). Further research exploring the strengths of parents with SMI and their children is warranted to inform family-focused strengths-based parenting interventions (50, 57, 58), reduce the stigma toward parental SMI (59), and increase self-esteem and coping skills (56).

Participants noted that parents with SMI may not present at services until in crisis and explored the barriers to seeking parenting and mental health support. Participants reported that the stigma of reduced parenting capacity generated a widespread fear of social service involvement and custody loss [e.g., (45)]. Professionals believed that parents therefore felt pressure to present as the “perfect parent” and were reluctant to seek support from services in fear of being deemed incapable of parenting. Previous research identified that delayed treatment for SMI predicts symptom progression, poorer social functioning, and reduced quality of life (60). Mental health professionals also believe that parents who are chronically unwell display higher levels of parenting need and likelihood of custody loss than parents experiencing a first episode psychosis receiving early intervention (37). Further research exploring the barriers faced by parents with SMI to accessing services is therefore required to increase the accessibility of services and enable early intervention.

Although recent research revealed that many adult mental health practitioners do not identify children of service users (20), most participants in this study believed it was important to consider the child's needs and all were able to discuss the impact of parental SMI on the child when prompted. However, consistent with previous research, there was conflict between prioritizing the needs of the parent and child (23, 24, 30, 37). Surprisingly, and in contrast to Tuck et al. (24), those who explicitly advocated for prioritizing the child's needs were from adult services. In line with the Think Family approach (26), most participants advocated for holistic working and family focused practice (FFP). However, research shows that in practice, many adult mental health professionals do not regard FFP (61) or the delivery of parenting interventions (47) to be part of their role. Previous research has identified barriers to implementing FFP including a lack of resources, confidence, and training to deliver such approaches (22, 62) as well as poor interagency collaboration (21). It would be beneficial for future research to comprehensively explore the variation in views across child and adult mental health practitioners to understand service specific barriers to FFP and subsequently provide recommendations for practice. Participants also identified the need for further training in parental SMI and the roles of other services to facilitate collaboration with other agencies and best meet the needs of the whole family. Further research is warranted to explore the training needs of UK professionals to inform improved, family-focused care for parents with SMI and their children. For example, recent efforts in the United States provide promising support for the feasibility of family-focused practice interventions with mental health practitioners to improve professional practice and parenting outcomes (63–65).

The findings from this study have further implications for clinical practice. Due to the risk of poor outcomes for parents with SMI and their children, professionals must receive training in a family focused approach to ensure early identification of the whole family's needs and subsequent early intervention. Professionals also require good service knowledge across adult and children's services to encourage signposting and referral to relevant services. For example, child and adolescent mental health services (CAMHS) in the UK, who are well-placed to support children adversely affected by parental mental illness. As parenting and mental health were believed to be intertwined, professionals highlighted the need for specialist services available to parents with SMI including a bespoke pathway tailored to the family's needs. Current services were deemed inaccessible and unacceptable to parents with SMI. Pragmatically, professionals should endeavor to encourage engagement with services by providing home visits and flexible appointment times to accommodate for childcare difficulties and other barriers to attending services. This study has provided a comprehensive overview of the needs of parents with SMI and their children and has the potential to subsequently inform the development of parenting interventions tailored to this population for which the evidence base is currently scarce (66).

### 4.1 Strengths and limitations

This is one of the first UK studies to explore both health and social care professionals' views toward parents with SMI. This study recruited a wide range of professional roles across NHS trusts and Local Authorities and was not limited to one geographical area due to the use of telephone interviews. We accessed the views and experiences of professionals working within adult and child services and provided an in-depth exploration of the needs of both parents with SMI and their children.

To ensure quality and rigor, the author engaged in reflexivity throughout the research and completed an audit trail of the analysis process. The authors conducted a comparison of preliminary independent coding to ensure codes were grounded in the data. However, not all quality assurance techniques used in template analysis were conducted (43). Due to pragmatic considerations, it was not feasible to conduct respondent feedback, although there is debate as to whether member checking enhances research credibility (67).

## 5 Conclusion

The findings from this study indicate that parents with SMI may experience complex parenting challenges in addition to those of other parents. Unpredictable and inconsistent parenting practices were viewed to have a largely detrimental impact on service user's children which highlights the importance of the early identification and provision of support to children. This study highlights a range of barriers to accessing services and demonstrates that current services do not adequately meet the needs of parents with SMI and their children. Many participants advocated for a whole family approach, however further training in parental SMI and improved collaboration between adult and child services is required to work holistically with families.

## Data availability statement

The raw data supporting the conclusions of this article will be made available by the authors, without undue reservation.

## Ethics statement

The studies involving humans were approved by University of Manchester Research Ethics Committee. The studies were conducted in accordance with the local legislation and institutional requirements. The participants provided their written informed consent to participate in this study.

## Author contributions

LO: Methodology, Writing – review & editing, Formal analysis, Writing – original draft. LW: Conceptualization, Data curation, Investigation, Project administration, Writing – review & editing. RD: Conceptualization, Supervision, Writing – review & editing. RC: Conceptualization, Supervision, Writing – review & editing. LG: Methodology, Writing – review & editing, Conceptualization, Supervision.
